# BayesPeak: Bayesian analysis of ChIP-seq data

**DOI:** 10.1186/1471-2105-10-299

**Published:** 2009-09-21

**Authors:** Christiana Spyrou, Rory Stark, Andy G Lynch, Simon Tavaré

**Affiliations:** 1 Statistical Laboratory, Centre for Mathematical Sciences, Wilberforce Road, Cambridge, UK; 2 DAMTP, Centre for Mathematical Sciences, Wilberforce Road, Cambridge, UK; 3 Cancer Research UK, Cambridge Research Institute, Li Ka Shing Centre, Robinson Way, Cambridge UK; 4 Department of Oncology, University of Cambridge, Li Ka Shing Centre, Robinson Way, Cambridge, UK

## Abstract

**Background:**

High-throughput sequencing technology has become popular and widely used to study protein and DNA interactions. Chromatin immunoprecipitation, followed by sequencing of the resulting samples, produces large amounts of data that can be used to map genomic features such as transcription factor binding sites and histone modifications.

**Methods:**

Our proposed statistical algorithm, BayesPeak, uses a fully Bayesian hidden Markov model to detect enriched locations in the genome. The structure accommodates the natural features of the Solexa/Illumina sequencing data and allows for overdispersion in the abundance of reads in different regions. Moreover, a control sample can be incorporated in the analysis to account for experimental and sequence biases. Markov chain Monte Carlo algorithms are applied to estimate the posterior distributions of the model parameters, and posterior probabilities are used to detect the sites of interest.

**Conclusion:**

We have presented a flexible approach for identifying peaks from ChIP-seq reads, suitable for use on both transcription factor binding and histone modification data. Our method estimates probabilities of enrichment that can be used in downstream analysis. The method is assessed using experimentally verified data and is shown to provide high-confidence calls with low false positive rates.

## Background

The importance of DNA-binding proteins in molecular functions such as transcription, replication, DNA repair and chromosome segregation highlights the significance of identifying the locations of their binding sites throughout the genome. The most widely used method for mapping these genomic locations is chromatin immunoprecipitation (ChIP). This process involves shearing the DNA and isolating the fragments to which proteins have bound [1], after which various methods can be used to identify those protein-bound fragments. A similar approach may be used to identify histone marks such as trimethylation. Direct sequencing is a reliable and efficient technique that is gradually replacing microarray hybridization for determining the contents of the immunoprecipitated samples [2]. These two procedures are widely known as ChIP-seq and ChIP-chip respectively, and both present their own statistical challenges. Hidden Markov models (HMM) fit naturally in this framework and have had numerous implementations in the analysis of ChIP-chip data sets [3-8]. However, these models are not directly applicable to ChIP-seq data.

In this paper we focus on a method of analyzing ChIP-seq data to identify protein-binding locations and the presence of specific histone modifications in the genome. Such data consist of the locations of the ends of the protein-bound and background fragments from the sample of interest as well as often containing control data from a sample that contains fragments of DNA with no preference for the regions to which the specific protein binds. Various statistical tools have been developed to interpret the data resulting from these techniques, but the set of available tools is not yet mature.

Our algorithm models the positions of the sequenced fragments and determines the locations of enriched areas, such as binding sites, by using HMMs and Bayesian statistical methodology. In this (and other) ways, it differs from previously published methods that we now briefly review.

ChipSeq Peak Finder [9] clusters the sequenced reads (*i.e.*, the beginnings of the fragments), and uses the ratio of the counts from the immunoprecipitated sample to the control in order to identify (or call) regions where large numbers of fragments overlap as "peaks". An updated version of the method, eRange [10], also allows the use of reads that map to multiple locations in the genome, which results in an increase in the amount of data applied to peak-calling.

The extended set method XSET [11] uses the full estimated length of the DNA fragments to identify the regions with the highest numbers of overlapping fragments. The method in Mikkelsen et al. [12] takes into account the "mappability" of the underlying sequence, by excluding regions from the reference genome that correspond to multiple occurrences of the same short sequences, and computes p-values to find significant differences between the observed and expected numbers of fragments. PeakSeq [13], another algorithm that allows for this mappability effect, starts with a normalization step comparing the control to the background component of the ChIP sample and then, using the Binomial distribution, identifies significantly different concentrations of reads between the two samples.

A feature of ChIP-seq is that, by examining only the start of protein-bound fragments, we can identify peaks offset on the forward and reverse strands of the DNA, the true binding site lying somewhere in between. Model-based Analysis for ChIP-seq (MACS) [14] shifts the reads on the forward and reverse strands together, and uses the Poisson distribution to identify the density of reads in enriched and non-enriched regions in order to call peaks. In addition, the method identifies multiple identical reads to avoid biases during amplification and sequencing library preparation.

Quantitative enrichment of sequence tags (QuEST) [15] also shifts the peaks from opposite strands together and derives a kernel density estimation score to call the enriched regions. FindPeaks [16] calls peaks according to some minimum height criteria without including a control sample in the analysis. Another algorithm is Site Identification from Short Sequence Reads (SISSR) [17], which estimates Poisson probabilities of high read counts, and calls regions where the peaks shift from the forward to the reverse strand.

In Kharchenko et al. [18] three similar peak calling methods are proposed that score read counts upstream and downstream of each region to match read patterns in the forward and reverse strands. In addition, Nix et al. [19] have simulated spike-in data, combined them with control reads from real experiments and used different metrics to score the peaks while controlling for false discoveries. Table 1 presents a structured comparison of these algorithms.

**Table 1:** Comparison of different peak-calling algorithms

**Method**	**A**	**B**	**C**	**D**	**E**	**F**	**G**
CSPF	control or IP only	read length no orientation	merge strands no shift	N	simple height criteria	ROC curve (empirically)	both

XSET	IP only	fragment length orientation	merge strands no shift	Y	simple height criteria	FDR estimate using Poisson distribution	both

Mikkelsen et al.	IP only	no orientation	no merge no shift	Y	*p*-values from permutations	no official FDR	both

MACS	control or IP only	fragment length orientation no duplicated reads	shift reads merge strands	N	Poisson *p*-values	FDR estimate by peaks in control:IP	both

QuEST	control	orientation	shift reads merge strands	N	kernel density estimation	FDR estimate by permutations of the control	better for TF

FindPeaks	IP only	fragment length orientation	no merge no shift	N	simple height criteria	FDR estimate by permutations of the IP	both

SISSR	control or IP only	fragment length orientation	no merge no shift	N	compares reads on different strands	FDR estimate by peaks in background:IP	better for TF

Kharchenko et al.	control	orientation	no merge no shift	N	Poisson distribution	FDR estimate by permutations of the control	better for TF

PeakSeq	control	fragment length orientation	merge strands	Y	sample normalisation Binomial distribution	FDR estimate, q-values (BH correction)	both

BayesPeak	control or IP only	fragment length orientation	no merge no shift	N	negative binomial distribution, Bayesian posterior probabilities	posterior enrichment probabilities	both

The methods shown are compared with respect to the following features:

A. whether they require a control sample (control) or whether they only use the ChIP sample (IP only)

B. whether they take into account the (average) length of the reads/fragments and their orientation

C. whether they take into account the different DNA strands or if they merge the reads, and whether the reads are shifted towards the 3' end

D. whether an externally estimated mappability file is used

E. how the scores, on which the classifications are based, are estimated

F. whether/how any FDR or sensitivity/specificity estimates are calculated

G. whether or not the method is applicable to both transcription factor (TF) and histone mark data.

## Results

### Algorithm

During chromatin immunoprecipitation, the proteins are cross-linked with the DNA, the cells are lysed, and the DNA is randomly sheared. The fragments bound by the protein of interest are isolated using specific antibodies to immunoprecipitate the protein and the cross-links of protein and DNA are reversed to liberate the DNA fragments. The resulting sample is enriched in the target immunoprecipitated areas but consists mainly of background DNA fragments.

Following the experiment, high throughput sequencing is used to reveal the identity of a sample of the fragments. The fragments are size-selected beforehand to improve the throughput and reproducibility of the sequencing reaction. We use the Illumina Genome Analyzer platform, in which the samples are placed on flow cells and go through several cycles of preparation, imaging and identification. The short reads from the ends of fragments are then mapped back to the reference genome to give the chromosomal position and strand of each read. The length of each fragment is unknown, since only one end is sequenced, but the average fragment length can be estimated experimentally (*e.g. *using the Bioanalyzer platform). In our analysis we only use the reads that map to a unique location of the genome.

An important part of the process is the addition of a control sample, such as an Input preparation, which undergoes the same cross-linking, fragmentation and sequencing procedure, the key difference being that the bound fragments are *not *isolated using an antibody. Our method can be applied with or without the inclusion of a control sample, but we would advise researchers to use control data, as they are necessary for identifying sequential artefacts or sample biases.

#### HMM model description

We have constructed a fully probabilistic model that takes into account the natural features of the data and incorporates them in a hidden Markov framework. The method divides the genome into equidistant regions, or windows, whose size is not less than half the mean fragment length; depending on the experiment and the length of the sites of interest, the resolution can be modified as desired. Counts for each window are defined as the number of 5' fragment ends (the end that was sequenced) that map to that region, either on the forward or the reverse DNA strand. We define these counts as  and  for window *t*, on the forward (+) and reverse (-) strand. The window length is chosen such that most fragments cover the window to which their 5' end is mapped and also the neighbouring one. Thus for windows *t *and *t *+ 1, the counts  and  have the same dependence on the underlying sequence.

We use a hidden Markov model (HMM) that assigns a state to each region *t *such that *S*_*t *_= 1, if there is a binding site or modification in that region increasing relative fragment abundance, and *S*_*t *_= 0 if not. We assume that the dependence between adjacent windows is the same throughout the genomic region under study, *i.e.*, *P*(*S*_*t*+1 _= 1|*S*_*t *_= 0) = *p *and *P *(*S*_*t*+1 _= 1|*S*_*t *_= 1) = *r *for parameters *p *and *r *and for all *t*.

**Figure 1:**
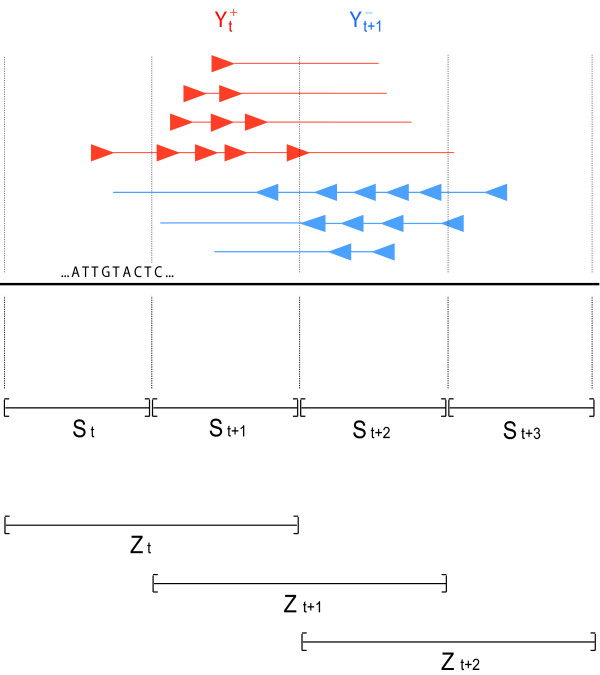
**Illustration of the model**. This figure shows how the reads (arrows) on the forward and reverse strand, indicated by red and blue respectively, are counted as  and  and depend on the nature of the underlying regions *t *and *t *+ 1 when their full length is taken into consideration. Moreover, this figure shows how each *Z*_*t *_state corresponds to the underlying ones *S*_*t *_and *S*_*t*+1_.

As the fragment counts  and  depend on both *S*_*t *_and *S*_*t*+1_, the working states correspond to the set of paired combinations



for all windows *t*. Figure 1 shows an illustration of the model. We consider states *Z*_*t *_= {1, 2, 3} to have the same enrichment effect and the state *Z*_*t *_= 0 to have none. The initial state distribution for *t *= 1 assigns equal probability to all 4 states.

Conditional on the parameters *γ *and *w*_*t *_(defined below) and on the hidden state *Z*_*t *_= 0, the counts are negative binomially (NB) distributed, and given *Z*_*t *_= 1 the counts are the sum of two independent negative binomial random variables, corresponding to background and foreground counts. Using this distribution avoids estimation problems caused by overdispersion of the data when greater variability than expected is observed. Such issues would arise if we used the simple Poisson model that implies equality between the mean and the variance of the counts. In addition, the NB can be expressed as a Poisson-Gamma mixture to make parameter estimation and additions to the model more straightforward. When a control sample is available, it is included in the analysis by introducing a parameter via Poisson regression, as shown below. In this way external factors causing high or low read concentration can be quantified using the density of the Input reads and protein-related enrichment can be correctly identified.

The emission distributions of the model are



where Γ (*α*, *β*) represents the Gamma distribution with density *f*(*x*|*α*, *β*) ∝ *x*^*α*-1 ^exp(-*βx*) for *x *≥ 0, *w*_*t *_is the number of Input fragments with 5' ends in windows *t *and *t *+ 1 (on either strand), *λ*_0 _and *λ*_1 _are the parameters corresponding to relative fragment abundance in the unenriched and enriched regions respectively, *γ *is the parameter that allows for the dependence on the Input sample, and *α*_0_, *β*_0_, *α*_1 _and *β*_1 _are hyperparameters.

#### Parameter and state estimation

Within the Bayesian framework of this paper we use efficient Markov chain Monte Carlo (MCMC) algorithms, as opposed to the Expectation-Maximization (EM) algorithm [20] that has been commonly used for HMM parameter estimation, thus providing a natural way of avoiding problems with unstable numerical optimisation. Bayesian methods estimate the model parameters by sampling from their full posterior density rather than giving point estimates, and offer the opportunity of including prior parameter information in the analysis [21].

MCMC algorithms take an approach similar to EM by using the complete data (*i.e.*, observed reads and missing hidden states) to sample from the posterior distributions of the parameters and states. The posterior samplers alternate between simulating the states given the parameters and read counts, and simulating the parameters given the complete data. We set Gamma priors for the parameters *α*_0_, *β*_0_, *α*_1 _and *β*_1 _and *γ *and Beta priors for *p *and *r*.

We evaluate the likelihood expression using a recursive technique introduced by Baum and Welch [22] that consists of forward and backward steps as also used in a Bayesian context by Scott [23]. Depending on the parameter involved at each step and whether it has a conjugate posterior distribution, the algorithm samples new values using either Gibbs or Metropolis-Hastings updates.

To sample from the posterior distribution of the states we use another recursive technique, called forward-backward (FB) Gibbs [23], which treats all the state parameters as one block, updates their distribution and then uses the FB recursions to sample each state directly from the joint density. This algorithm leads to more rapidly mixing runs, since the Markov chain consists of fewer components and the dependence of each hidden state on its previous drawn value is reduced.

The nature of the hidden states is then estimated using their marginal posterior probabilities, which indicate whether each window is enriched or not, according to the model and the data. More details on how these are calculated can be found in the Methods section. Once the posterior probabilities for the ***Z ***states are estimated, it is very easy to calculate the equivalent ones for the ***S ***states that correspond directly to the existence or absence of a site of interest in each window. In our examples we chose a natural threshold of 0.5 so that the peaks that we called are those regions that are more likely to be enriched than non-enriched. In other circumstances, we might choose a different threshold to return only highly probable regions, or conversely more speculative regions. In our examples, the majority of posterior probabilities are near to either zero or one, and so the exact choice of threshold would have little effect.

## Implementation

We applied the method to ChIP-seq data to study both transcription factor binding and histone modification assays. We used ChIP samples for the liver-enriched transcription factor HNF4*α *and trimethylated lysine 4 histone 3 (H3K4me3) from livers of mice primarily of the Black 6 strain [24,25]. In addition, a control sample was used, consisting of Input DNA that went through the same crosslinking and shearing process but without immunoprecipitation.

The reads were aligned using Illumina's Eland program, allowing for up to two mismatches in the first 32 bases of each read. To improve efficiency of the algorithm we split the genome into 5 Mb-long regions and analysed them separately using non-overlapping windows. Furthermore, we ran the method twice, the second time using an offset of half a window's length, to classify correctly all the regions and avoid any bias due to the position of the windows. We then merged all the called peaks between the two runs.

### Choice of window length

**Figure 2:**
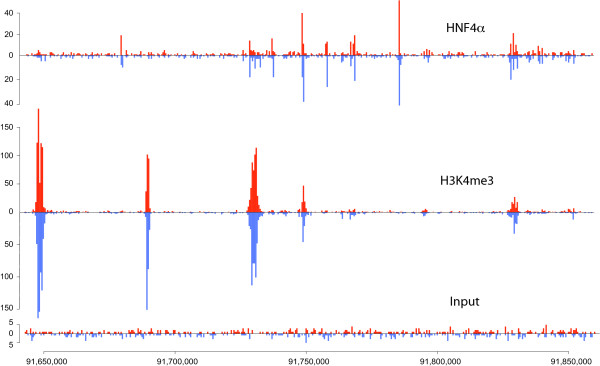
**A view of the HNF4*α*, H3K4me3 and Input data**. This diagram shows the read distribution on a region of chromosome 16 for the HNF4*α*, H3K4me3 and Input samples. The red plots represent the reads mapped to the forward strand and the blue correspond to the ones mapped to the reverse strand. We can see that HNF4*α *has short, sharp peaks and noise in the background regions, whereas H3K4me3 has peaks that are much longer and contain most of the sample fragments since there are very few of them in the non-enriched areas.

**Figure 3:**
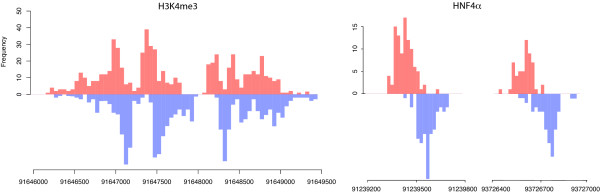
**A closer view of some HeK4me3 and HNF4*α *peaks**. These histograms present the counts of the 5' ends of the reads from the H3K4me3 and the HNF4*α *data, forming peaks on the forward (red) and reverse (blue) strand. The offset between them shows how the enclosed area corresponds to an enriched region. The plots are on a different scale to show the density of reads clearly and highlight the difference between the peaks formed by a histone mark and a transcription factor.

As expected, the transcription factor and histone mark data look very different. The first have narrow peaks at the locations of the binding sites, and the latter have wider enriched regions that are usually covered by multiple peaks. Figure 2 shows a genomic region of the two data sets and the control sample highlighting the differences between them, and Figure 3 shows some enriched regions for HNF4*α *and H3K4me3 respectively and how they consist of individual or multiple peaks.

To ensure the algorithm is suitable for both analyses, we applied it using different window-lengths. The length of the library fragments as reported by the experimental procedure was in the range 110-260 bp with a slight preference for shorter fragments. The mean fragment length of approximately 190 bp places a lower limit on the window size of a little under 100 bp, and our desire for fine resolution places an upper limit of approximately 300 bp on the window size, for which reason we investigated window sizes of 100 bp, 200 bp and 300 bp.

We observed that, as the length of the windows increased, fewer enriched regions were identified for both samples. For example, for HNF4*α*, the model with 100 bp windows called 22 more peaks than the one with 300 bp windows, and for H3K4me3 the respective comparison resulted in 9 additional peaks. We believe that the model with shorter windows was the best one to use, since it identified the largest number of peaks. These regions were tested for agreement with other algorithms, and validated using motif analysis and visual comparisons, as explained in the next sections.

### Inclusion of the control sample

Our method can be implemented in a manner that makes use of a control sample or not, as the situation dictates. For the HNF4*α *and H3K4me3 data sets, where a control was available, we also ran the model ignoring those control data. As anticipated, the presence of control data improved the results. We observed that in the absence of a control sample, our technique did not identify some peaks that were identified when the control sample was available, specifically 17% fewer peaks for the HNF4*α *sample and 2% for H3K4me3. One might anticipate that the purpose of the control was to prevent the calling of false positive peaks, and so be surprised by this result. However, in our model the lack of information leads to greater unexplained variance, and the distinction between background and foreground is less obvious.

### Checking adequacy of the model

Since several assumptions have been used in the construction of the algorithm, it is important to check the practical fit of the model. In a classical setting, a goodness-of-fit test compares observed and fitted values by quantifying how extreme the data are if we assume the model to be true. In a Bayesian framework, model fit can be tested using posterior predictive distributions of test statistics that can be functions of both the data and the parameters [26]. These statistics, also called *discrepancy variables*, emphasize the goal of assessing the discrepancy between model and data, as opposed to testing the model's correctness.

To investigate relevant features of ChIP-seq data, some sensible discrepancy variables include the mean number of reads in each window, the corresponding standard deviation, and the maximum possible number of reads in one location. We plot histograms of the simulated values, which represent the posterior predictive distributions, and visually compare them to the observed values.

**Figure 4:**
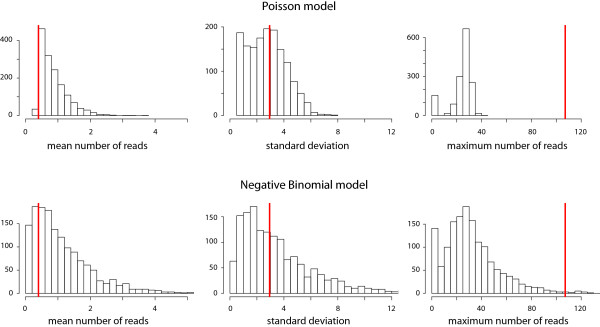
**Checking the model fit: Histograms of the discrepancy statistics for the HNF4*α *data**. The red lines represent the value we observe from the data for the mean number of peaks per window, their standard deviation, and the maximum possible read score. The histograms are plots of the same variables estimated using the 3,000 simulated values of the parameters during the run of the algorithm. The closer the real value is to the simulated distribution, the better the model explains those aspects of the data. The first row shows the three histograms of the values generated using the Poisson model and the second row shows the corresponding values for the negative binomial model. The latter shows a notable improvement in explaining these features of the data.

Since the Poisson distribution has been widely used in modelling the distribution of reads in the genome in ChIP-seq applications, we compare it with the more flexible model we propose. In Figure 4 we present plots of discrepancy variables generated from a model using Poisson emission distributions (without the Gamma mixture shown in previous sections) as well as the negative binomial model that we have used to produce our results. From the histograms we see that, for the Poisson model, the distribution of the 3,000 simulated values for the maximum number of counts in each window fails to accommodate the observed value (observations are shown as red lines). This is a clear sign that the Poisson model cannot deal with overdispersion in the data. In addition, the observed average number of reads per window for the Poisson model lies closer to the tail of the distribution, compared to the negative binomial. The Poisson distribution allows for less variability and thus may have a larger mean to accommodate some of the higher observed values. There is little evidence, on the other hand, suggesting discrepancy between the negative binomial model and the data. This strengthens our belief that the more flexible model is desirable to better explain the important features of the data.

#### Testing

##### Application to exampled data

**Figure 5:**
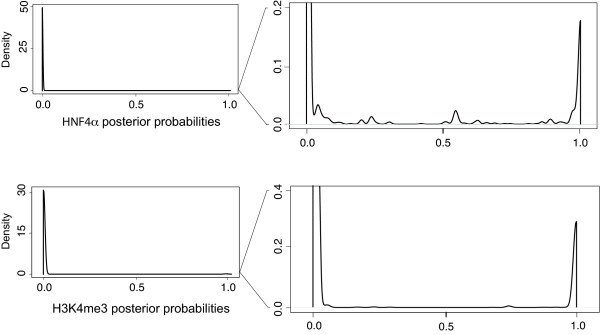
**Posterior probability plots**. These plots show the distributions of the posterior probabilities *P*(*S*_*t *_= 1), *i.e.*, the probability that there is a binding site at window *t*. On the left are the plots of the probabilities for the HNF4*α *and H3K4me3 data sets shown in full scale, and on the right are the same plots with a closer view of the tails of the distributions. We can see that the vast majority of the regions have zero probability of being enriched and the remaining regions have probabilities very close to 1. Only a handful of regions lie in the middle of the range, and these are likely to lie in the tails of large peaks. To include some of these regions, we set the threshold to be equal to a posterior probability of 0.50 and called every region with a probability greater than that as enriched.

We present the results from region 90,000,000-95,000,000 bp on mouse chromosome 16, which accommodates a large number of reads and is likely to have more sites than a randomly selected genomic region. As can be seen in Figure 5, the posterior probabilities of enrichment from our model are for the most part practically 0 (most windows show no evidence of enrichment), while most of the remaining probabilities are very close to one (those windows that show strong evidence of enrichment and will be in regions we call as peaks). We set a threshold at 0.50 for both data sets and classify all regions with probabilities greater than that as enriched.

Our method called 149 peaks for HNF4*α *and 58 for H3K4me3 and we compared these results with the findings of other peak-callers. The second time the algorithm was run, using an offset of half a window's length, we identified two additional peaks compared to the initial run, for both data sets, which implies that it would be unlikely to identify any more regions with a third shift of the window boundaries.

**Figure 6:**
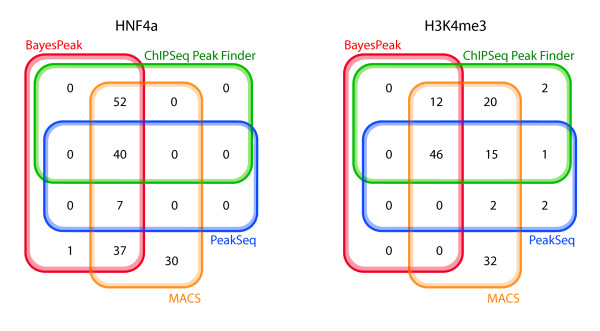
**Algorithm comparison for HNF4*α *and H3K4me3**. This figure shows how the peaks called by BayesPeak, ChipSeq Peak Finder, MACS and PeakSeq compare. For CSPF we used a maximum gap of 100 bp between reads to create clusters, which were then identified as peaks if they contained more than 9 reads, more than 5 of which overlapped at the same base, and if the total count was larger than the corresponding number in the Input sample by a factor of 5. To identify the enriched regions using the other methods, we chose p-values smaller than 0.05 for both MACS and PeakSeq. The number of peaks called by BayesPeak seems smaller in these diagrams than we report in the paper because in some cases more than one region is covered by a long peak identified by another method; in this case, they are merged into a single peak covering the entire region.

We used the algorithms ChIPSeq Peak-Finder (CSPF), MACS and PeakSeq for comparison. We chose those three because they take different approaches and vary in complexity, thus giving a wide spectrum of possible results. CSPF uses simple height criteria in comparison with the control sample to call peaks, whereas MACS takes a model-based approach by first scanning for peaks on opposite DNA strands and then using Poisson probabilities to detect enrichment. As mentioned in the introduction, PeakSeq takes into account the mappability of the underlying regions and makes the control and ChIP samples comparable by normalising the first with respect to the background noise of the latter. Then it uses binomial probabilities to generate p-values and detect significantly large read concentrations. The results are presented as Venn diagrams in Figure 6.

In our examples, we note that all but one of the peaks called by BayesPeak are identified by at least one other method, thus giving us confidence that BayesPeak is calling only true peaks. Peaks that other methods call, but that BayesPeak does not, may show discrepancy from the model that underpins BayesPeak, as will be discussed in the conclusions. MACS reports more peaks than any other method, suggesting that MACS may be returning peaks that represent false positives. Note that BayesPeak can return more peaks by accepting a lower posterior probability threshold, but these additional peaks are more likely to be background features.

##### Motif analysis

Transcription factors bind to a set of specific DNA sequences, many of which have been identified by previous studies. We used motif analysis to check whether the called peaks contain the known motif of the transcription factor, indicating that they represent proper transcription factor binding sites.

We searched for the HNF4*α *motif in each individual peak by using the mouse-specific Positional Weight Matrix (PWM) as previously published [27]. We then calculated the score for the best possible match in each peak and estimated the probability of this score occurring by chance. To do so we used 1,000 permutations of the bases of each sequence to simulate other sequences, calculated the maximum enrichment score for each one and then found the proportion of these scores that were larger than the score of the true sequence.

**Figure 7:**
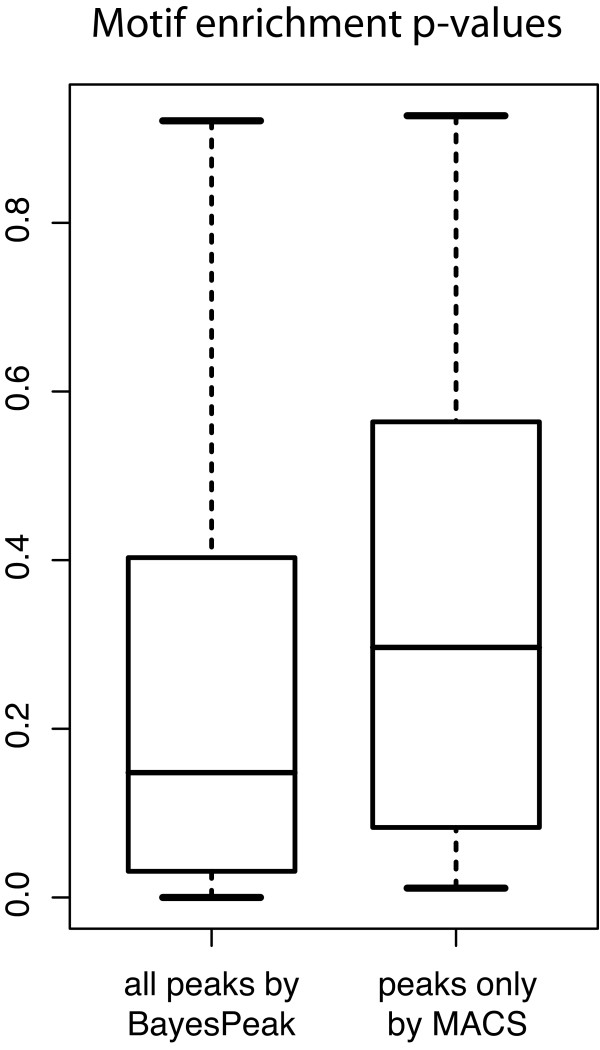
**Box-plots of motif enrichment p-values**. We grouped all the sequences of the regions called by BayesPeak (including the ones called by MACS) and the ones detected only by MACS. Here we compare boxplots for the groups of called peaks, which summarize the p-values corresponding to the significance of the motif enrichment in each of the respective sequences. We observe that the regions identified by BayesPeak give lower p-values, on average, compared to the ones called by MACS only, strengthening the indication that enrichment is less likely to have occurred by chance and that binding does take place at those sites. A separate analysis with CLOVER used on the same groups, yielded p-values of less than 10^-6 ^for the regions called by BayesPeak and greater than 0.10 for those identified only by MACS.

We can interpret these proportions as p-values, and the smaller they are, the greater the evidence that the peaks contain the transcription factor binding sequence. In Figure 7, boxplots of these p-values are displayed for the groups corresponding to all the regions called by BayesPeak (including ones also called by MACS) and the peaks identified by MACS only. We observe that the regions identified by BayesPeak give lower p-values compared to the other group, which suggests that the motif appears more often in the peaks we do call than in the candidate regions we miss.

In addition, we took another approach to check for over-representation of the motifs in the same two groups of enriched regions. For that purpose we used the program CLOVER [28], which uses a library of possible motifs for different transcription factors (in this case, the JASPAR CORE library [29]) and tests whether for any of them the groups show an unusually large distribution of high enrichment scores. The peaks called by BayesPeak were significantly enriched for the HNF4*α *motif with a p-value less than 10^-6^, and no other of the available motifs was detected. In addition, the program did not report significant enrichment of any transcription factor motifs for the regions identified only by MACS, as the p-values for the corresponding tests were larger than 0.10. According to these findings, the regions identified by BayesPeak, most of which also called by MACS, have stronger evidence for binding than the ones that are uniquely identified by MACS.

##### Validation data sets

The objective testing of ChIP-seq peak calling methods is somewhat challenging, since spike-in data sets cannot yet be generated due to the difficulty of replicating the nature of the data, and simulated data sets do not give a close match to an experimental outcome. However there are two small data sets for which enriched and background regions have been validated, and we apply our methods to these.

The first such set that we consider was presented by Johnson et al. [9] on the Neuron-Restrictive Silencer Factor (NRSF/REST). The data set includes 83 *in vivo *binding sites defined by ChIP-qPCR (quantitative real-time fluorescence Polymerase Chain Reaction) and 30 sites that equivalently showed no enrichment [30].

We ran BayesPeak on their unamplified data and used the posterior probability threshold of 0.50 to call peaks. (The majority of identified peaks had probabilities greater than 0.98, as was also the case with the HNF4*α *and H3K4me3 peaks identified previously.) We called 74 peaks out of the 83 validated positive regions and zero of the regions that were confirmed negative, thus achieving sensitivity 89% and specificity 100%.

**Figure 8:**
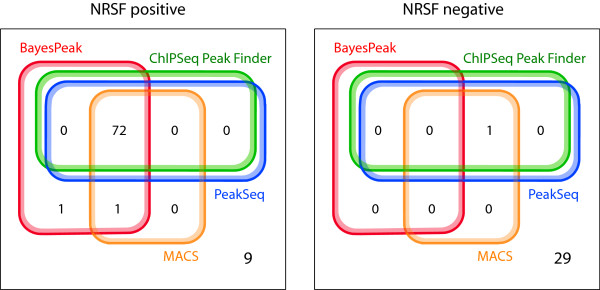
**Algorithm comparison for NRSF data**. This figure shows how the known-positive and known-negative regions called by the same four methods compare. We merged those results for CSPF and PeakSeq that were identical.

In Figure 8 we compare our results to the corresponding results of the other peak-callers and observe that out of the 74 positive regions that were identified by any method, 72 were called by all of them. Similarly, 9 regions were not identified by any peak-caller. BayesPeak detected the largest number of enriched regions and none of the known-negatives, whereas the other methods incorrectly called one negative region.

As a final test, we analysed another transcription factor data set, namely Tal1 (also known as Scl), with a set of 24 experimentally-validated enriched regions [31]. The regions bound by Tal1 had been tested in transgenic mice where all regions function as tissue-specific regulatory elements. This data set was analysed without including a control sample in the model to confirm that the known true positive regions are still identified.

We ran BayesPeak using the Tal1 sample only and identified the enriched regions using the same posterior probability threshold of 0.50 as before. Our method did find all 24 enriched regions, 20 of them having posterior probabilities greater than 0.90. Comparing with the other algorithms as before, CSPF also gave the full list of known positives, whereas PeakSeq could not be used due to the absence of a control sample. MACS did not make any of the expected calls and reported a problem in building its model.

## Discussion

A lot of binding peaks are obvious and will be identified by any sensible method. This, coupled with the small amount of available validated data, makes it difficult to show definitively that one method is better than another. We have however shown that our method performs well, and that it has several qualities that make it attractive for inclusion in an analysis suite.

The key advantages of our model, that other methods do not offer, are the Bayesian approach to parameter and state estimation, and the use of the negative binomial distribution. Within the Bayesian paradigm, we are returning posterior probabilities as our measure of certainty. Therefore we do not call regions as enriched simply because they do not look like background, but only if they look more like a peak than background. This offers the greatest scope for interpretation, as well as allowing for the use of probabilities as weights in subsequent analyses (*i.e.*, motif discovery).

After submitting this manuscript, we became aware of another Bayesian implementation [32] of HMMs to both ChIP-chip and ChIP-seq data. Our application is different in terms of the features of the model, the emission distributions and the treatment of the control sample.

The negative binomial distribution allows for overdispersion and provides a better fit to the data than the Poisson distribution that has been widely used by other methods. Not only did the Poisson distribution appear inferior in the comparison of discrepancy variables, but peak calling methods that incorporate it failed to identify some peaks in both the HNF4*α *and H3K4me3 data sets.

Other features of our method that are desirable, but shared to some degree by some other methods, are the accounting for strandedness and orientation of the fragments and the ability to identify binding regions more precisely. Accounting for strandedness is essential for modelling the peaks, but our use of this information differs from that of previous methods. BayesPeak also tends to identify narrower regions than other methods, which is a particular advantage when searching for transcription factor binding sites.

**Figure 9:**
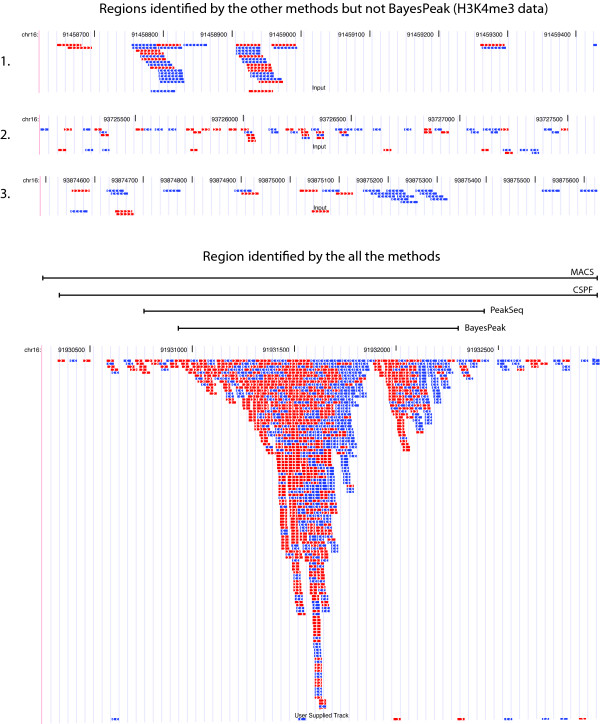
**Some peaks called only by the other three methods and an example of a peak called by BayesPeak**. In this figure, there are three regions (labelled 1, 2, 3) that BayesPeak did not identify as peaks that the three other algorithms did. The plots show the first 32 bases of each fragment, the ones corresponding to the forward strand in red and the reverse strand in blue. In addition, we include an image of a peak that was called by our method to show how the shape compares with the others. The images were generated using the UCSC genome browser.

Peaks identified by BayesPeak appear to be less likely to contain false positives. The high-confidence regions identified by our method tend also to be called by other methods. Additionally, the motif analysis we carried out showed high enrichment for the transcription factor's binding sequence. However, for the H3K4me3 data set, there were peaks that the other methods call, but that ours does not; we present examples of those in Figure 9, contrasted with a typical high-probability enriched region. Regions one and three show departure from background, but do not conform to what we expect to be the appearance of a peak. In the first example, there are a large number of reads, but their relative orientations do not match our expectation (*i.e.*, that the positive strands should lie to the left of the reverse strands), while in the third region only the reverse strand shows evidence of enrichment. The second illustrated region shows very low-density reads, but there are enough for some methods to call enrichment.

The other advantage of the modelling framework that we have used is the ability to adapt and extend the model as required. More states could be introduced to cope with a different physical model of fragment length to binding-site length, and simple modifications could allow for the use of paired end data. Finally, it is becoming more common for the locations of multiple transcription factor binding sites and locations of histone modifications to be investigated together for the same sample. The tendency is for each to be compared separately to a control sample, and due to financial and experimental pressures the same control sample tends to be used for each - a situation that is clearly not ideal. Our approach can be extended to model all samples simultaneously, sharing information about background levels and preventing the inappropriate over-influence of the control sample.

## Conclusion

We have presented a flexible and adaptable method for the detection of enriched regions of the genome that offers advantages over methods currently in use and performs well for those few data sets with validated peaks.

## Methods

### Availability

The code is available from our website [33], with some instructions and data.

### Algorithm description

1. The genomic region is divided into windows and the data are converted into numbers of reads per window for each sample.

2. Starting values are assigned to the parameters of the model.

3 i. The likelihood is calculated recursively using forward and backward variables; the information is updated with every new state and the distribution of states is updated using the observed data and the likely transitions.

The forward variable can be defined as  where ***θ ***= (*p*, *r*, *α*_0_, *β*_0_, *λ*_0_, *α*_1_, *β*_1_, *λ*_1_, *γ*), which subsequently gives the forward recursion for *i *= 0, 1, 2, 3



where  and *q*_*ji *_= *P *(*Z*_*t*+1 _= *i|Z*_*t *_= *j*). The backward variable is defined by , which gives the backward recursion for *i*



Then, the likelihood can be represented by



and the probability that the state of window *t *takes the value *i*, given all the data ***Y***, is



The probability that *S*_*t *_= 1, *i.e.*, that there is a binding site in region *t*, is equal to *P *(*Z*_*t *_= 2) + *P *(*Z*_*t *_= 3). To prevent underflow, we use the normalisation constant *c*_*t*_^-1 ^= Σ_*j*_*α*_*t*_(*j*) and scale the terms to *α*'_*t*_(*i*) = *c*_*t*_*α*_*t*_(*i*), *β*'_*t*_(*i*) = *c*_*t*_*β*_*t*_(*i*), which does not change the recursions.

3 ii. The states are then simulated from the distribution *p*(*Z*_(1, *T*)_|*Y*_(1, *T*)_, ***θ***), which is the joint posterior mass function of all the states given ***θ***. A simple expression can be used to calculate *p*(*Z*_(1, *t*)_|*Y*_(1, *t*)_, ***θ***) recursively for *t *= 1,..., *T *and then, starting from *Z*_*T*_, each state can be drawn in a backward simulation from *p*(*Z*_*t*_|*Y*_(1, *T*)_, *Z*_(*t*+1, *T*)_, ***θ***) for *t *= *T *- 1,..., 1. Details on the state posterior density and the forward-backward Gibbs sampler can be found in [23].

4. Given the complete data set (observed read counts and simulated states), each parameter is updated conditionally on the values of the remaining parameters using Gibbs updates. For most of them the form of the likelihood and the conjugate priors lead to closed-form posterior distributions such as Beta for *p*, *r *and Gamma for *β*_0_, *β*_1_, *λ*_0 _and *λ*_1_. As this is not the case for *α*_0_, *α*_1 _and *γ*, we use Metropolis-Hastings updates with symmetric (Normal) proposals centred at their accepted values.

5. Steps 3 and 4 are repeated a number of times and the updated values of the model parameters and state probabilities are recorded at each simulation. Averages of those probabilities give estimates of how likely the states are to take the values 0, 1, 2 or 3 and since any region *t *must be either 2 or 3 to contain a binding site *Z*_*t*_, the significance score for each region is equal to the sum of those two probabilities.

### State estimation and classification

Given the Gibbs draws of the parameters {***θ***^(1)^,..., ***θ***^(*m*)^} and the corresponding hidden states {***Z***^(1)^,..., ***Z***^(*m*)^}, where for the *j*^*th *^draw ***θ***^(*j*) ^= {*p*^(*j*)^, *r*^(*j*)^, *α*_0_^(*j*)^, *β*_0_^(*j*)^, *λ*_0_^(*j*)^, *α*_1_^(*j*)^, *β*_1_^(*j*)^, *λ*_1_^(*j*)^, *γ*^(*j*)^} and ***Z***^(*j*) ^= {*Z*_1_^(*j*)^,..., *Z*_*T*-1_^(*j*)^}, our aim is to estimate the marginal posterior distributions of the states defined by *π*_*t*_'(*i*) = *P *(*Z*_*t *_= *i*|***Y***) and decide on their classification. The obvious estimator would be



which can be improved by using expectations of the terms to become



where *π*_*t*_'(*Z*|***θ***^(*j*)^) is just the posterior probability calculated for the *j*^*th *^simulation of the MCMC algorithm. This process introduces a layer of Monte Carlo variability, since it averages probabilities rather than events simulated with those probabilities.

### Prior distributions

According to the transition probabilities of our model, *p *corresponds to the probability that a 100 bp window is part of an enriched region, given that the region to the left of it is not enriched. Thus it represents the probability that an enriched region appears as we move along the genome. *r *corresponds to the probability that, given an enriched region, the window to the right of it is also enriched. Therefore it is related to the expected length of the sites. Since we do not have any information on the number or length of the bound regions, we set these two parameters to have a symmetric distribution around 0.5. The *γ *parameter reflects the relationship between the abundance of reads in the control and the ChIP sample. We set this parameter to have a mean value of 0.5 and a long tail to accommodate possible large values.

For the remaining parameters, we used more informative prior distributions, based on the nature of the enriched and background regions. We expected very few reads to map to regions where no binding is taking place, thus *λ*_0 _should be close to zero. On the other hand, enriched areas have a wide range of fragment concentrations, reflecting the different binding affinities of the protein; therefore *λ*_1 _should be allowed to vary considerably. We controlled for these features by tuning the scale and rate parameters of the Gamma priors of the hyperparameters *α*_0_, *β*_0_, *α*_1 _and *β*_1_.

More specifically, for both datasets we used the prior distributions



where a Beta(*α*, *β*) distributed variable *x *has density *f*(*x*|*α*, *β*) ∝ *x*^*α*-1^(1 - *x*)^*β*-1 ^for *x *∈ [0, 1].

### Implementation details

The algorithm was coded in C, and Perl was used to pre-process the data and do some of the motif analyses. The current implementation of the code analyses 5 Mb at a time. This allows for the parallelization of a genome-wide or chromosome-wide analysis and saves the need for either a) making genome-wide assumptions regarding the consistency of parameters or b) adding complexity to the model. The code can be altered to change the length of sequence being examined, however the current implementation is unlikely to scale to genome-wide within typical computational constraints. Other implementations of the model would be possible if this were desired.

**Figure 10:**
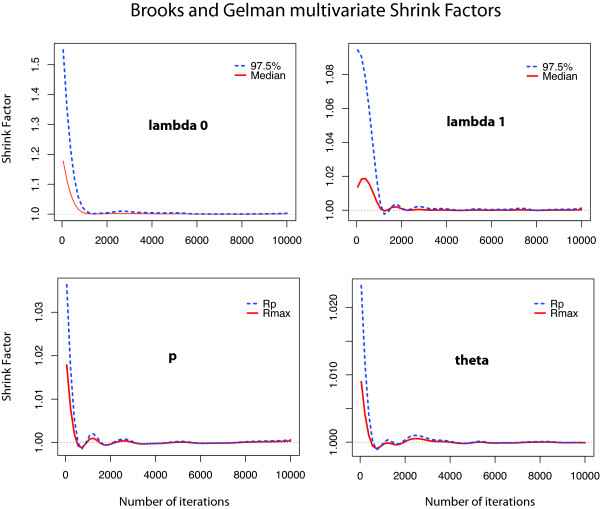
**Checking for convergence of the MCMC chains: Plots of the Brooks and Gelman shrink factor**. These plots show how some of the parameters of the model from different runs of the algorithm (on HNF4*α *data) reach their stationary distribution after burn-in and therefore the simulated values we use are indeed samples from the posterior distribution of interest. The plotted factors show whether the chains have mixed well by comparing the variance of the simulated values within and between different chains.

We ran the MCMC algorithms for 100,000 iterations, thinning the chains and saving every 10th value to avoid correlation between consecutive draws and save computational space. To use the updated values as samples from the posterior distribution, we made sure that the values had reached stationarity and that the number of simulations was large enough to represent adequately the full range of the distribution. To do so, we ran three chains starting with different parameter values, discarded the first 2,000 values from the thinned sample and applied some formal diagnostic tools that check for convergence and good mixing between parallel chains. The Bayesian Output Analysis package (BOA) [20] in R provided a range of these diagnostics that test for stationarity of functions of the parameters and compare different posterior samples. We used the Brooks, Gelman and Rubin test [34] that uses parallel chains with different initial values to test whether they all converge to the same target distribution. It compares the variance within each chain and the variance between chains to check how similar they are. In Figure 10 we plot the Brooks and Gelman shrink factor for some of the parameters to demonstrate how it reaches 1 after about 2,000 iterations (which we allowed for burn-in), thus showing agreement between the different chains. Furthermore, we used the Geweke [35] diagnostic and the Heidelberger-Welch stationarity test [36] that test whether single chains have reached equilibrium by comparing means from the early and latter parts and checking whether the chain comes from a covariance stationary process. Our simulations passed the tests successfully, suggesting that the chains had reached the stationary distribution and were run for long enough.

## Authors' contributions

CS developed and implemented the model. RS compared the results between the different methods. AGL and ST supervised statistical aspects of the model. All authors drafted and approved the final manuscript.
